# Update on the neurodevelopmental theory of depression: is there any ‘unconscious code’?

**DOI:** 10.1007/s43440-020-00202-2

**Published:** 2020-12-31

**Authors:** Małgorzata Gałecka, Katarzyna Bliźniewska-Kowalska, Michael Maes, Kuan-Pin Su, Piotr Gałecki

**Affiliations:** 1 Department of Psychotherapy, Medical University of Lodz, Aleksandrowska 159, 91-229 Lodz, Poland; 2 Department of Adult Psychiatry, Medical University of Lodz, Lodz, Poland; 3 Department of Psychiatry, Faculty of Medicine, Chulalongkorn University, Bangkok, Thailand; 4 An-Nan Hospital, China Medical University, Tainan, Taiwan

**Keywords:** Depression, Proinflammatory cytokines, Th 17 cells, Regulatory T cells (treg), Allostasis, Neurodevelopmental

## Abstract

Depression is currently one of the most common psychiatric disorders and the number of patients receiving antidepressant treatment is increasing every year. Therefore, it is essential to understand the underlying mechanisms that are associated with higher prevalence of depression. The main component leading to the change in functioning, in the form of apathy, anhedonia, lack of motivation and sleep disturbances, is stress. This is the factor that in recent decades—due to the civilization speed, dynamic technological development as well as competitiveness and competition in relationships—significantly affects the psychophysical condition, which results in an increase in the prevalence of civilization diseases, including depression. To understand the mechanism of susceptibility to this disease, one should consider the significant role of the interaction between immune and nervous systems. Their joint development from the moment of conception is a matrix of later predispositions, both associated with the mobilization of the proinflammatory pathways (TNFα, IL-1β, IL-6) and associated with psychological coping with stress. Such an early development period is associated with epigenetic processes that are strongly marked in prenatal development up to 1 year of age and determinate the characteristic phenotype for various forms of pathology, including depression. Regarding the inflammatory hypothesis of depression, interleukin 17 (IL-17), among other proinflammatory cytokines, might play an important role in the development of depressive disorders. It is secreted by Th17 cells, crossed the placental barrier and acts on the brain structures of the fetus by increasing IL-17 receptor levels and affecting the intensity of its signaling in the brain.

## Introduction

Depression is currently one of the most recognized disease entities in the world. Its prevalence is increasing every year and is a serious medical, social, and economic problem [1]. Recent studies show that the tendency to depressive episodes can be formed in very early childhood, even in utero, and has an impact on the further formation of brain structures [2]. This influence, according to recent studies, is associated with epigenetic processes largely mediated by the immune system [3]. Interest and development of research related to the joint influence of the development of the immune and nervous system may be of significant importance in the prevention and treatment of depression, especially of the drug-resistant nature.

## Modulating factors: what influences the development of depression?

The factor that affects long-term and recurrent depressive episodes is stress, i.e. a change in the physical or mental condition of the body, caused by the action of potentially threatening stimuli. Physical stressors can be, e.g. hunger, illness, injury, fatigue, and psychological stressors, e.g. loss of loved one, grief, too high demands of the environment, lack of a sense of closeness. Studies related to the analysis of the impact of stress on our lives show that the vulnerability to depression might be increased due to increased inflammatory reactivity [4–6]. Studies related to the observation of a group of adolescents showed that a greater exposure to stressful interpersonal life events was correlated with a significant increase in depression over time, but only in people showing faster TNFα mobilization and interleukin-1β reactivity to social stress [7]. Depending on the stimulus, the characteristics of the stress response may vary, but it always has a specific purpose. It should cause such changes in the functioning of the body that will allow adaptation to new environmental conditions and, as a result, regain balance. Immune changes caused by stress are initially beneficial because they allow the body to adapt to individual needs, but in the long run they can have a harmful effect and cause the development of diseases in adulthood [8, 9].

It happens that the body adapts to functioning in a depressive mode, and through epigenetic changes this tendency is passed on to subsequent generations [10]. An important feature of body’s allostatic adaptation is the short duration of its protective effect. In longer periods, with persistent allostasis, it exerts a detrimental effect. If the release of stress hormones is not completed, and thus the effect on target cells is prolonged, there is a decrease in receptor sensitivity (down regulation phenomenon) and damage to tissues and organs, which must bear the cost of ineffective response of the body [11]. Long-term stress leads to dysregulation and causes excessive intensification of the stimulating glutaminergic transmission and glutamic acid level in the brain, which leads to the excitotoxic death of neurons [12]. Allostasis, i.e. the ability to maintain the body’s balance by changing its functioning, in some cases helps to survive. It happens, however, that in certain situations it leads to a reduction of physical and mental needs and, as a result, the development of a depressive state. It is also connected with a change in the way the immune system works.

An important element is the impact from the very conception of changes taking place in the body at very early stages of life, shaping a predisposition to an increased depressive reaction in the future.

## First brain programming

Studies indicate that the first months of fetal life are an important element in the development of susceptibility to depression in adulthood. The specific period of the neurogenesis process lasts throughout the pregnancy and the first year of life of the child. In the prenatal period, the embryo and fetus develop rapidly, and many neural pathways are formed. During these periods of special sensitivity (the so-called critical windows), the effects of certain adverse factors may be most pronounced [13]. Strong chronic stress during pregnancy has been proven to inhibit neurogenesis [14], lead to neuronal atrophy, and consequently causes anatomical and pathophysiological changes in the brain of the unborn child. Maternal cytokines and glucocorticosteroids can penetrate the fetus and affect the development of the descendant’s brain, by interfering with cell differentiation, axonal growth, and synaptic connectivity, and by reducing the total hippocampus volume. Increased prenatal cytokine level may also disturb amygdala growth, while glucocorticosteroids increase the number of neurons and microglia [15].

## Impact of maternal immune activation: IL-6 and IL-17

The psychophysical state of the mother is important in the development of a child’s predisposition to develop depressive disorders in the future. Although the body coordinates and compensates to a large extent its actions, it can react in a way that affects the fetus in situations of excessive overload. Cytokines, signaling molecules for the immune system that are generated, i.e. in response to infection or strong and chronic stress, are crucial in activating the mother’s immune system and can cross the placenta. The cytokine known in this aspect is IL-6; it has an impact on brain development and behavior of the offspring [16]. An important element in the development of a predisposition to depression in offspring is the increase in IL-17 plasma levels in mothers before conception and during pregnancy. Interleukin 17 level can be raised not only due to an ongoing autoimmune disease in mother, but also due to depression. Studies confirm that people with recurrent, particularly drug-resistant depression have an increase in IL-17, which can become a marker of diagnosis in depression that does not respond to antidepressant treatment [17, 18]. Activation of the mother’s immune system from infection or autoimmune predisposition leads to an increase of IL-6 serum level, which in turn activates Th17 cells to produce IL-17. This interleukin crosses the placenta and acts on the brain structures of the offspring by increasing levels for the IL-17 receptor, further increasing IL-17 signaling in the fetal brain [19]. This sequence of events is required for maternal immune activation (MIA) to cause fetal cortical changes [20]. This view is known in the development of three basic behavioral abnormalities associated with the autism spectrum (ASD) and the development of autoimmune diseases in offspring [21]. However, taking into account the inflammatory theory of depression, IL-17 also plays an important role in the development of civilization diseases, including a significant one in depressive disorders.

In vitro studies on mouse Th17 cells indicate that transforming growth factor-beta (TGF-β) and IL-6 are necessary for their formation from undifferentiated CD4 + T cells. Without the participation of IL-6 they develop towards regulatory T cells (Treg) in connection with the activation of the transcription factor Foxp3 [22]. As a result, Th lymphocytes, in particular Th17, which are co-responsible for the body’s immunity, redirect their activity, affecting the development of autoimmune diseases and depression [23, 24]. In addition, the significant increase in IL-6 associated with the mother’s strong stress and the increase in cortisol in the mother’s body in the fetal period has an impact on the subsequent development of the child on the differentiation of CD4 + T cells into Th17 inflammation lymphocytes [25]. Therefore, an important element in the neurodevelopmental nature of depressive disorders is the psychophysical state of the mother and her ability to cope with immune activation in connection with environmental and disease factors. The efficiency of mothers’ immune responses as well as their ability to cope with stress is an important factor modulating the future competences of their offspring in regulating mood and coping with stress.

## The fetal immune system and the development of depression

Until recently, scientists have argued to what extent the immune system can affect a child’s development in prenatal life. Studies have confirmed that antigen-presenting cells are present in the skin, spleen, thymus, and lungs of the fetus from the 13th week of pregnancy. In the in vitro test, they were able to respond to bacterial and viral antigens, stimulating T cells, which is similar to adults. Dendritic cells play an important role in the differentiation of Treg cells, which are involved in the inhibition of increased immune responses [26].

Prenatal exposure to maternal immune activation activates microglia [27, 28], which affects the subsequent increase in the activity of a wide range of cytokines, growth factors and free radicals. The long-term impact of changes in the future may contribute to elevated levels of neurotoxic inflammatory mediators in adults. Increased oxidative stress in pregnant women who are in a disadvantaged socio-demographic situation and have greater psychological stress in pregnancy, as the research shows, is associated with more complications during pregnancy and childbirth. Oxidative stress is a mediator of maternal psychological stress on the fetus [29].

An important aspect is the fetal development stages. Research mainly on the mouse model confirms the occurrence of critical moments for the development of predispositions for future disorders. There are no clear studies and answers as to their exact course. The factor modulating the response is the duration of the maternal immune response. Cytokines and other signaling molecules affect the forming brain of the fetus. Animal studies confirm that the later disturbances in utero occur due to maternal immune activation (MIA), the less important this is for the brain of the developing fetus [30]. Studies in a mouse model found that rodents exposed to lipopolysaccharide (LPS)-induced maternal immune activation in the mid-fetal period had an increased apoptosis at the dentate gyrus after birth compared to the risk factor group at the end of fetal life. Thus, it can be concluded that gestational age at the time of exposure is a critical determinant of changes in dopaminergic-driven behaviors, like motor and reward-seeking alterations, in further functioning [31]. In the fetus, it is assumed that the risk of increased impact decreases after the first trimester. Regarding mood disorders, it is reasonable to recognize the period to about half of the pregnancy due to the formation of the hippocampus structures between 11 and 20 weeks of pregnancy [32]. Hippocampus, as a part of a limbic system involved in regulation of emotions [33], is a cortisol-sensitive brain structure [34]. In male fetuses, cortisol associated with the activation of the pituitary–hypothalamic–adrenal (HPA) axis, enters the bloodstream to a lesser extent, which may be important in the sexual dimorphism in the occurrence of depressive disorders [35]. There is a strong evidence for sexual dimorphism in associations between prenatal maternal stress and infant neural development and temperament, with girls appearing to be at elevated risk for profiles linked to anxiety and affective disorders [36]. However, the impact of fetal stress factors should be understood as the possibility in later life of developing predispositions in a demanding environment. The fetal immune challenge is a starter for some changes. Factors that determine changes in the phenotype of an individual’s functioning and are closely related to genetic and environmental factors.

An important element in the development of the fetus, apart from the immunological and disease-related ones, is also environmental factors associated with maternal nutrition and stress during pregnancy [37, 38]. Studies on the mouse model confirm that a high-fat diet results in a smaller number of social interactions in offspring, reluctance to explore novelty [39]. These behavioral changes corresponded to anatomical changes in the paraventricular nuclei (PVN) of the hypothalamus, with a reduced number of cells producing oxytocin, a neuropeptide that had previously proved to be important in modulating social behavior [40]. Similarly, when it comes to stress, its effect on the immune system is scientifically recognized fact [41]. Regardless of multifactorial origin, stress, systemic inflammation, and immunization coincide in effects on offspring [42, 43].

There is a connection between stress and immunological response. The connecting element is the hypothalamus–pituitary–adrenal axis (HPA axis), which coordinates behavioral actions—‘fight or fly’. Anxiety and depressive behavior characterize offspring exposed to prenatal stress in rodents. The behavioral phenotype is associated with changes in HPA axis regulation in both mother and offspring [44]. Glucocorticoids as selective antagonists [44] and corticotrophin releasing factor affect T and B cell proliferation, NK cell cytotoxicity and cytokine receptor density [45]. Fetal stress also affects the activity and expression of 11β-hydroxysteroid dehydrogenase type 2 in the placenta, which converts glucocorticoids into inactive metabolites that limit the transfer [46]. Hsiao and Patterson have shown that maternal placental immune cells are responsible for the growth of IL-6 in the fetus. Genetic inhibition of the ability of stem cells to induce IL-6 protected the behavioral phenotype in the offspring [47].

## Early childhood: environmental impact

The field study involved several-month-old children of healthy mothers and women with depression during pregnancy and after delivery. It turned out that babies recognize emotions much faster than objects in their surroundings. Their reactions to their mothers’ face frozen for a few minutes were observed. The children of depressed women did not react in any way to this, while the babies from the control group became restless, irritable, began to cry, thus forcing some kind of reaction of the environment, because these sensations were unnatural for them [48]. Field research shows that children are adapted to a certain way of functioning from the very beginning of their lives. Anxiety is a symptom of helplessness, which prompts to try to tune in, get an adequate response. Its absence causes chronic stress, which in turn can cause a depressive reaction, being nothing but an attempt to reduce anxiety, maintain homeostasis by adapting to a sense of lack of influence, powerlessness.

In the first year of a child’s life, in addition to developing the functions of individual systems, the development of the emotional and social sphere is extremely important. Creating bond is a key aspect of development of major importance for the further life of a person. The quality of the child’s early emotional bonds and their impact on further social and emotional functioning are important for understanding various irregularities in children and adolescents in the context of the development of behavioral disorders, aggression and depression related to the processes of emotional regulation.

Modern knowledge regarding the formation of bonds and consequences for the emotional and social development of a person largely uses the assumptions of the attachment theory formulated by John Bowlby. He described attachment as an instinctive, evolutionary bond that plays an important role in the survival of the species. It develops in the first year of life and has an impact on the relationships of the individual [49, 50]. Insufficient satisfaction of the need for closeness in the early stages of a child’s life (e.g. due to social isolation, low parenting skills, emotional rejection of parents) leads to changes in the neurobehavioral response to experienced stressors, which shapes our future relationships with people [51]. Lack of an adequate response from the guardian means that subsequent unsuccessful attempts to satisfy (crying, screaming) the child finds too aggravating, which shapes his passive attitude. At the same time, there is a dysregulation of the endocrine and immune systems through a network of mutual feedback in the HPA axis. Persistent personality traits in the form of anxiety mindset, through dysregulation of the HPA axis, are in turn a source of constant proinflammatory activity of the immune system [52]. In addition, the lack of an adequate response or lack of response from the environment, caregivers at all, affects the development of anxiety in the early stages of child development. In this case, the HPA axis also sends at relatively low loads, an unusually large number of signals to increase the stress hormone in the blood. This creates greater susceptibility to stress, strengthens the sense of anxiety and aggression, and also affects developing cognitive functions [53].

Studies on both humans and animals confirm the significant role of attachment. Separation from the mother at an early stage of the child’s life leads to permanent neuroendocrine changes, manifested in adulthood in the form of cognitive, emotional, and social deficits [54]. Mother’s postpartum depression and the degree of anxiety experienced by her as a permanent personality trait were associated with the quality of the neuropsychological development of the children [55]. Importantly, the mother’s mental state is associated with the state of her proinflammatory activity. Studies conducted on mothers and their preschool children have confirmed that mothers showing more emotional warmth and attention to children have lower C-reactive protein (CRP) values and lower resting potential of the sympathetic nervous system. Also, children of these mothers had lower parameters of the variables studied (CRP, resting potential of the sympathetic nervous system), which confirms the impact of attachment styles and the impact of upbringing on the development of proinflammatory predispositions [56].

Due to the joint development of the nervous and immune systems, the impact is bi-directional. At the early stages of development, the dysregulation caused by the body’s stress, both biological and mental, is an important factor modulating the interaction of both systems [57]. The direction can be both strengthening and decreasing future coping competences and regulation of the interaction of both systems and active reverse inhibition of the HPA axis, which results in inhibition of further glucocorticoid release from the adrenal glands.

Linking a confident attachment relationship with the development of individual brain structures and mechanisms of neurohormonal regulation at early stages of development is an important element in the further development of personality factors, especially those associated with anxiety and strongly associated with depression in adulthood. A secure attachment style is linked with direct, clear communication of emotions, empathy giving the opportunity to take into account the perspective of the second person. Positive emotions dominate and negative ones are usually a signal of frustration or conflict, but they are characterized by transience [58]. When there is a lack of such features of relation, it is characterized as the type of anxiety-ambivalent bond, avoidant or disorganized, and influences lower competences in the future in regulating emotions and coping with stress, as well as personality vulnerability to depression [59]. From the period of late pregnancy to the second year of life, intense reorganization changes occur in the brain, especially in the right hemisphere, which is responsible for the development of attachment function [60]. This process involves the creation and consolidation of new synaptic connections, the death of unused and myelination of nerve cells. Connections that arise during this period (including the right hippocampus and the right supraorbital lobe) are directly related to the experiences in the relationship with the mother [61]. It has been shown that the development of a child’s brain occurs by leaps and bounds, and the first clear developmental leaps occur at 2 and 8 months of age. At around 8 weeks of age, the child acquires the ability to process visual information in such a way that it can read the mimic emotional expression of the guardian. The interaction between the mother and the child, by looking into each other’s eyes, according to research results, stimulates the development of connections between the right frontal cortex and the limbic system (amygdala) [61]. Therefore, the mother’s abilities and competences have a significant impact on further neuronal, immunological, and behavioral development.

## Epigenetics, or inheritance of memory ‘unconscious code’

Research conducted in the last 20 years clearly confirm the impact of epigenetic modifications in the development of various types of disorders, including depression [62–65]. Prenatal development and early childhood development are important here, as researchers emphasize [38, 66]. They are an important developmental period during which, despite a specific genotype, plasticity occurs in the form of reprogramming the expression of the child’s genes referred to as different genomic methylation patterns [67]. This is particularly important in developmental periods of the formation of nervous system structures, personality formation and stress susceptibility, which will be responsible in the future for regulating emotions and coping strategies. In addition, it is assumed that early life stress affects the occurrence of brain and peripheral changes that can ultimately affect behavior through epigenetic mechanisms such as DNA methylation, histone modification and microDNA processing [10, 67]. Epigenetic processes are important in relation to the availability of genetic information at the cytokine gene loci in CD4 + T cells, whose presence, as confirmed by research, has an impact on the development of depression in later years [24]. Studies on mice confirm that regulatory cell dysfunction (Treg) affects the susceptibility of mice to the development of depression-like behaviors during inducing stress to a greater extent than in wild-type mice—in the unburdened group [68].

These results can confirm that the dysfunction of these cells in drug-resistant depression can be not only a consequence of the disease, but also an important aspect in its development, which is affected by epigenetic processes. In addition, in a study on mice lacking Treg cells, significantly positive correlations were observed in 5-HT and its metabolite, 5-HIAA, present in the hippocampus, which showed high glucocorticoid receptor (GC) levels [69]. Visible decreases in 5-HT levels were observed in mice deficient in CD4 + CD25 + Treg cells. In vitro studies of Th17 mouse cells show that transforming growth factor beta (TGF-β) and IL-6 are required to form from undifferentiated CD4 + lymphocytes [22]. As a result of co-stimulation with these two cytokines, STAT3, RORa, and especially RORgt proteins are activated—the main transcription factors causing lymphocyte differentiation towards Th17 [70, 71]. In the case of stimulation of undifferentiated lymphocytes only by TGF-β in high concentration, without the participation of IL-6, they develop towards regulatory cells (Treg) as a result of activation of the transcription factor Foxp3 [22, 72]. The concentration of interleukin 6-acute phase protein with pleiotropic immunostimulatory activity is, therefore, a factor determining the fate of undifferentiated CD4 + cells [73]. In our opinion, this is important in shaping the vulnerability to developing depression in adulthood. Early stages through epigenetic influence affect subsequent changes in the form of an increase in proinflammatory cytokines, which determines the formation and maintenance of the Th17 phenotype which is important in the development of drug-resistant depression in adulthood.

Recent studies also confirm the epigenetic interactions responsible for the onset of depression. Traumatic experiences cause long-term changes in DNA methylation, with the consequence that the system responsible for regulating stress hormone/cortisol expression is deregulated. This reduces the ability to cope with stressful situations, which causes people with mutations, e.g. in the FKBP5 gene, who more often suffer from depression, post-traumatic stress and anxiety disorders. It has been proved that the mentioned modifications can be permanent [74, 75].

Evidence for the impact of epigenetic changes are, among others work of the team of Prof. Rachel Yehudy from Israel, researching survivors of the Holocaust. Physiological changes were noted in the children of people who experienced high stress in their childhood or youth. Rachel Yehuda and her team studied the intergenerational effects of trauma. Scholars have shown that the descendants of Holocaust survivors have a different stress hormone profile than their peers. It cannot be ruled out that they are predisposed to disorders related to the feeling of fear. They found that survivors of the Holocaust have more stress hormone. Lower cortisol levels were noted. It is not known why there was less cortisol and cortisol-degrading enzyme. It also turned out that the younger a person was during the Holocaust, the less cortisol was found in this person’s body [76–78].

During subsequent studies, not only those who lived during World War II, but their children were examined. It turned out that, like their parents, many had reduced cortisol levels. However, unlike parents, they had higher levels of cortisol-degrading hormone than usual. Scientists speculate that this is the result of an adaptive mechanism in utero. Usually, this hormone is present in large quantities in the placenta and its task is to protect the fetus against maternal cortisol. If the placenta was low in hormone, and yet we know that its level in Holocaust survivors is reduced, too much cortisol could get into the fetus, as a result of which, it alone produced large amounts of the protective hormone. As Yehuda explains, epigenetic changes are designed to prepare a child to live in an environment similar to that in which parents live. However, in this case, the needs of the child from utero outweighed the influence of the parents’ environment. The children of those who survived the Holocaust, having little cortisol in the body and a lot of the enzyme that breaks it down, are less suited to living in their environment than their peers. This may be the reason why the descendants of survivors are more likely to develop post-traumatic stress disorder (PTSD) [78, 79].

Also in a study conducted on a group of early school-age children who were diagnosed with depression in the prenatal period, a reduced methylation of the glucocorticoid receptor (NR3C1) gene, mineralocorticoid receptor (NR3C2) gene and the serotonin receptor (SLC6A4) gene were indicated. Genes associated with the HPA axis were selected in this study [80].

Studies related to transgenerational transmission of trauma confirm that apart from genes we also inherit the memory of anxiety [81]. This fact may be the basis for generational changes, especially in relation to civilization diseases, among others metabolic disorders such as obesity, hypertension, diabetes, and depression, which are associated with stress.

Epigenetics then becomes our ‘unconscious code’. The code that is transmitted outside of genes and its representation are changes, inter alia in the form of a predisposition to depression, anxiety memory, which we consciously do not code, but our body through epigenetic mechanisms does.

## Depression: a mental representation of changes in the body

Studies confirm that depressed individuals have reduced levels of regulatory T cells (Treg), as well as increased levels of interleukin 17 (IL-17) [17, 18] and interleukin 6 (IL-6), which stimulate proinflammatory processes. This is associated with an increased stress response and cortisol secretion. This confirms the impact of immune responses on the development of depressive disorders. This condition can be compared to an autoimmune process in which the body’s immune system destroys its own cells and tissues. In depression, due to the constant elevated concentration of proinflammatory interleukins in the body, there is a weakening of the cognitive functioning of patients, and as a consequence may cause neurodegenerative changes, and later also dementia. During the study of pilots and anti-terrorists, it turned out that individuals with a more resilient personality negatively correlated with markers of the inflammatory process (IL-1, IL-6, CRP), and thus coped better with stress [82]. It is worth emphasizing that knowledge about the immunological basis for the development of depression opens new therapeutic possibilities.

An important element in the neuroimmunological background is also the fact that CD4 + T cells in patients diagnosed with depression are subject to spontaneous accelerated apoptosis [83, 84]. Test results, in both humans and laboratory animals, confirm that during chronic stress, T cell apoptosis increases [85]. The explanation for this phenomenon, especially in connection with the increase in immune activation, is tryptophan depletion. In the context of T cell apoptosis, tryptophan is the primary proliferative stimulus for T effector cells, and in a tryptophan-free environment, T cells will undergo apoptosis [86, 87].

Studies show that people with depression significantly increase the risk of neurodegenerative diseases, among others Alzheimer’s disease, Parkinson’s disease or multiple sclerosis as a result of oxidative stress associated with increased activity of reactive oxygen species (ROS) [88], insufficient activity of antioxidative defense mechanisms of the body and the occurrence of central inflammatory reactions. The importance of these factors in the formation of mild cognitive impairment is also emphasized [89].

In addition, chronic stress and established personality traits with anxiety attitudes, through dysregulation of the HPA axis, are a source of constant proinflammatory activity of the immune system [52]. The cascade of mutual feedback, due to excessive production of neurotoxic compounds (mainly so-called tryptophan catabolites—TRYCATs), gradually leads to the neurodegenerative processes described earlier (hyperactivity of limbic structures associated with reduced prefrontal cortical inhibition), manifested, among others in the form of depression [90, 91].

## Learning to feel safe: can changes be reversed?

The abilities of developing structures are important in future coping and perception of the surrounding world. Brain plasticity, however, allows to compensate for many changes, especially in early childhood. It depends on favorable conditions, intimacy, and emotional support.

Both epigenetic and immunological changes, at some stage of life, can be reversible, e.g. through a proper educational process, reducing stress and anxiety. It is important to have a proper response from the environment to the signals sent by the child. Hugging, comforting a child, or meeting their needs can turn off the HPA axis, which releases the stress hormone. In this way, the young body ‘learns’ a sense of security. On the other hand, high levels of stress in early childhood and a lack of response to basic needs can cause permanent stimulation of the HPA axis and the development of its hypersensitivity. The increase in reactivity means that over time, even a small stress stimulus will cause a significantly stronger reaction, which in adult life can lead to the development of depressive states.

The quality of the relationship with the caregiver, and how it alleviates the emotional state of the baby, has an impact on the creation of self-regulation capacity, i.e. the ability to regain balance in changing environmental conditions. Emotional regulation processes are an important protective mechanism. The relationship between mother and child has a positive effect on the development of the child’s self-regulation function if the mother shows sensitivity (responsiveness), i.e. a certain degree of openness to the child and its needs. This sensitivity also refers to the readiness to respond not only to negative, but also to positive reactions of the child.

In studies on children, it has been documented that the child-caregiver relationship is crucial from both a vulnerability and protective function perspective. Long-term studies were conducted on a large group of children, in which due to perinatal complications, one third of the respondents were included in the ‘high risk’ group. The results of the study show that children who have many risk factors do well as long as these factors are balanced by adequate resources in the form of protective factors (adequate care, support system) [92]. In the study of children’s life stories, given for adoption, whose mothers were diagnosed with mental disorders, it was shown that well-functioning adoption families completely balanced the genetic burden, and disturbed relationships in adoptive families caused more mental disorders in children compared to adopted children without genetic susceptibility [93].

Studies confirm that the mother’s emotional competence has a lasting impact on the child’s neuropsychological development from birth and even conception. Children of depressive mothers in adult life more often suffer from anxiety-depressive disorder. Factors that are particularly important for the development of cognitive, emotional, and social deficits are emotionally and physically difficult events for the child, such as the loss of a loved one, mother’s depression or illness. Supplying these emotional states allows you to alleviate the effects of stress and regain a sense of security in the child. For mothers with diagnosed depressive disorders, special support allowing for satisfactory contact with the child and increasing the mother’s sense of competence in this respect is essential. This confirms that calm, balanced mental development in childhood, especially up to the age of 1, gives a strong foundation for coping with stress in the future. Therefore, the experience of adequate care and developmental stimulation is important for further healthy development.

## ‘Unconscious code’: summary

In our opinion, the common process of developing the nervous and immune systems is crucial for the development of predisposition in adult life in the form of various forms of pathology, including depression. So what is the ‘unconscious code’ in the development of depressive disorders? It can be said that it is memory that works at the cellular level. During our development, cells become different from one another by changing their genetic program in response to transient stimuli. Long after the stimulus is gone, "cellular memory" mechanisms enable cells to remember their chosen fate over many cell divisions [94].

**Fig. 1:**
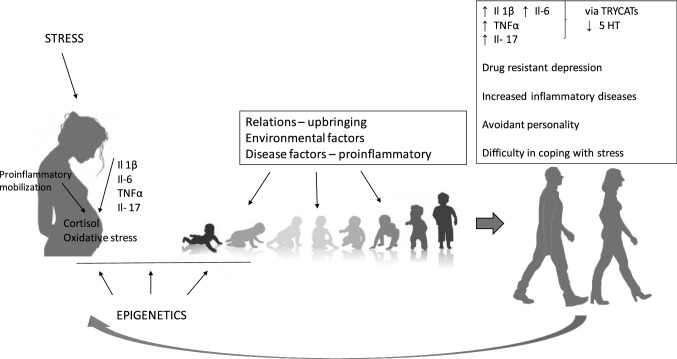
Maternal cytokines activated in connection with stress or the disease process have an impact on the future development of the fetus and predisposition to disease, especially in the aspect of civilization diseases, including depression. In the future, they result in increased proinflammatory activation in adulthood. Epigenetic processes play a key role

The body is able to ‘remember’ its states using epigenetic processes [95]. In the future, this shapes the development of the body’s adaptive capabilities. The activation of the mother’s immune system has a special role, in our opinion. Chronic stress as well as autoimmune and civilization diseases, increase of which we observe in the following years, have an impact on the growth of IL-6 and, consequently, IL-17, which mediate the fetus sensitizing it to possible cytotoxic factors. Then the ‘saved’ predisposition can be activated later in life in adverse environmental conditions. The body has less adaptability in the biological context, but also in the emotional–behavioral phenotype. Consequently, if developmentally it was associated with emerging centers of emotion regulation, it affects the increase of continuous experienced anxiety visible in the personality structure [96]. Anxiety as a constant personality trait is an important predisposing factor of a depressive episode. Just as pain is a symptom and an adaptive mechanism in physical ailments, anxiety has a similar function in mental and behavioral representation. When it appears, it is a signal for a change. The development of depression occurs when chronic ailments activate a cascade of ‘epigenetically recorded’ possibilities. In such a situation, the body accepts those associated with passivity and withdrawal as more adaptive. The ‘coded’ effect associated with proinflammation activates and maintains a cascade of biological (IL-6, IL-17, TNF, TRYCATs) and psychological (anxiety) events acting on a feedback principle and contributing to drug resistance in depression (Fig. 1).

An important element is therefore the neurodevelopmental nature of depression, as well as the search for new directions of treatment through immunological interaction. Currently, there are biological drugs in the research process that are used in autoimmune diseases with a strong inflammatory background, e.g. psoriasis or rheumatoid arthritis, but studies confirm that they also improve patients’ mood [97]. In addition, two-way impact in the treatment process is important, both biological and psychotherapeutic, related to realizing the possibilities and limitations as well as self-regulation skills of the patient.
